# Massive Ameloblastoma Requiring Awake Nasal Fiberoptic Intubation

**DOI:** 10.7759/cureus.40760

**Published:** 2023-06-21

**Authors:** Nafisa Chowdhury, Joaquin A Cagliani, Andre Loyola, Joseph SchianodiCola

**Affiliations:** 1 Anesthesiology, State University of New York (SUNY) Downstate Health Sciences University, Brooklyn, USA; 2 Department of Anesthesiology, Brookdale Hospital Medical Center, Brooklyn, USA

**Keywords:** nasal intubation, respiratory distress, intraoral mass, awake fiberoptic intubation, ameloblastoma

## Abstract

Ameloblastomas are rare tumors that arises from the odontogenic epithelium. Although benign and slow growing, an extensive lesion may cause airway obstruction, making bag-mask ventilation and intubation a significant challenge. Here, we present a 54-year-old male in respiratory distress with an 18x15x13 cm submandibular mass causing airway compromise. The tumor was extensive, occupying most of the oral cavity. Unable to perform direct laryngoscopy because of the tumor burden, we performed an awake nasal fiberoptic intubation to secure the airway. Successful intubation was achieved as well as subsequently tracheostomy. We subsequently provide a discussion on associated challenges and management options for patients with ameloblastomas.

## Introduction

Ameloblastomas are benign and locally aggressive neoplasms that grow from odontogenic epithelium in the maxillofacial area [1,2]. They account for 1% of all oral tumors and about 9-11% of odontogenic tumors, making them the second most common odontogenic tumors after odontomas [1,3].

These patients commonly present with face swelling, dental malocclusion, pain, and paresthesia of the affected area [4-7]. Given their anatomical proximity to the larynx, they can cause partial or complete airway obstruction creating an emergency scenario [8].

Most ameloblastomas are managed conservatively with a good prognosis by surgical enucleation [1,4]. Some ameloblastomas can grow to an extensive size and cause gross deformities and regional dysfunction [9]. These lesions are termed “giant ameloblastomas.”

Here we discuss a patient with a giant ameloblastoma who underwent successful, emergent anesthetic management. Additional discussions on associated challenges and management options are included in subsequent sections.

## Case presentation

**Figure 1:**
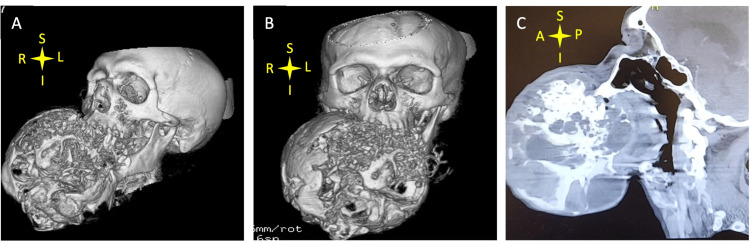
3D reconstruction of imaging (A-B); CT scan in sagittal view identifying 18x15x13cm submandibular mass (C). A: anterior; P: posterior; S: superior; I: inferior; L: left; R: right

Our patient was a 54-year-old male with a large mandibular tumor resulting in respiratory distress. On physical exam, the patient was alert and oriented. His American Society of Anesthesiologists (ASA) classification was 3E due to the emergent nature of the condition. On airway assessment, patient was determined to have a difficult airway with a Mallampati score of 4. Due to the presence of the large lesion, an upper bite test was unable to be performed. Vital signs included: blood pressure 100/66, heart rate 126, oxygen saturation 100% on nasal cannula at 3 L/min with non-distressed spontaneous ventilation. On imaging, CT scan of the neck showed an 18x15x13 cm heterogenous soft tissue tumor arising from the right mandibular ramus occupying most of the oral cavity and causing airway compromise (Figure 1). Given the emergent nature of this presentation, an emergent intubation was planned to secure the airway. A multi-team approach was used with otolaryngologists on stand-by for emergency tracheostomy prior to intubation in case of failure to access the airway through intubation. Due to the tumor size, direct laryngoscopy was not possible. Therefore, we performed an awake nasal fiberoptic intubation.

**Figure 2:**
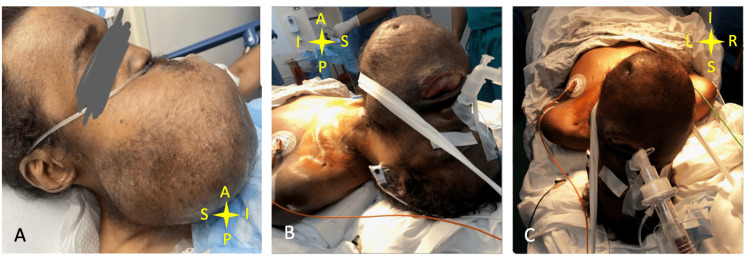
Picture of patient before and after awake nasal fiberoptic intubation A: anterior; P: posterior; S: superior; I: inferior; L: left; R: right

The patient was transported to the operating room bed where he was preoxygenated and pretreated with 2 mg of midazolam and 0.2 mg of glycopyrrolate intravenously. A phenylephrine nasal spray was used in each nostril to vasoconstrict the nasal vessels. To suppress the gag reflex, Cetacaine topical anesthetic was sprayed into the posterior glossopharyngeal area. The patient was also given 10 mL of Xylocaine Viscous (a 4% Lidocaine hydrochloride solution) and was instructed to gargle the solution and swallow. The patient was unable to gargle due to the tumor but was able to swallow. Using 2% Lidocaine jelly, serial upscaling nasopharyngeal airways (30, 32, 34, 36 French) were introduced into each of the nares to identify the more patent nostril and help dilate. The patient was then sedated with 10 mg of ketamine. To block laryngeal and tracheal stimulation, the patient received a transtracheal injection of 4 mL of 2% Lidocaine. A lubricated and pre-warmed 6.5 mm endotracheal tube (ETT) was inserted into the right nostril and then advanced with fiberoptic intubation via ETT. At that time, there was significant mass effect causing the epiglottis to be edematous and pushed posteriorly. Additional maneuvering was required to insert the fiberoptic scope between the epiglottis and the posterior wall. The vocal cords were then visualized, and the scope was passed into the trachea. The ETT was passed over the fiberoptic scope into the trachea. Its position was confirmed through the fiberoptic camera, and the tip of the endotracheal tube was positioned 2 cm above the carina. End tidal CO2 was confirmed by capnography before securing the endotracheal tube (Figure 2). Anesthesia was deepened using propofol and fentanyl, while rocuronium was used as an adjuvant for controlled ventilation. Anesthesia was then maintained with sevoflurane.

Once the airway was secured, the surgeons decided to start with surgical tracheostomy in anticipation of prolonged intubation and multiple follow-up surgical procedures and to avoid any risk of airway compromise. During the tracheostomy and retraction of the ETT, the fiberoptic scope was reinserted to act as an exchange ETT bougie and removed once the tracheostomy tube was inserted and confirmed. The patient tolerated the procedure well with an uneventful surgical and anesthetic course.

Post-procedure, the patient remained on the ventilator while recovering in the ICU. However, the patient presented with severe malnutrition and his hospital course was complicated by pneumonia resulting in severe right pleural effusions requiring thoracocentesis. The patient later developed severe sepsis and expired on hospital day #33.

This may be the largest case of ameloblastoma documented, as the largest ameloblastoma seen on recent database search was measured 15.2x11.4x12.0 cm [9].

## Discussion

Ameloblastomas are odontogenic tumors that arise in the jaw. Because of their aggressive growth and potential for large size, they present anesthetic concerns such as airway obstruction and difficult mask ventilation and tracheal intubation. They can distort the facial contour making mask ventilation and adequate jaw thrusts difficult or impossible [10]. The intraoral extension of the lesion can also cause airway obstruction and worsen the visualization of the glottis [10]. Given the edematous intraoral cavity, direct laryngoscopy may result in trauma causing bleeding which further obscures the view and results in aspiration of blood [10]. Tracheal intubation of these patients carries high complication rates ranging from transient hypoxia and hypotension to cardiovascular collapse and death. Finally, even though the patient may not have any symptoms of airway obstruction in the awake state, symptoms can present under anesthesia with muscle relaxation [10]. Therefore, a comprehensive review and management of the patient’s airway is essential.

Here we have described a case of successful intubation in a patient presenting with a large ameloblastoma and associated risk factors. We achieved a secure airway using the awake fiberoptic intubation technique. Given the extreme nature of the patient’s anatomy, using a laryngoscope or laryngeal mask airway (LMA) would have been impossible, particularly because these methods would cause bleeding and obstruct the laryngeal view.

Awake fiberoptic intubation is often indicated when there is anticipated or suspected difficulty in airway management due to abnormal anatomy and altered airway patency (i.e. head and neck tumors), cervical spine instability, and risk for aspiration or inability to tolerate a period of apnea [11,12]. The advantages include keeping the patient spontaneously breathing, improving visualization of vocal cords, and reducing the stimulation of the sympathetic response compared to direct laryngoscopy [13,14]. All these advantages allow for better hemodynamic stability. Contraindications to this technique include copious secretions or bleeding, local anesthetic allergy or sensitivity, ongoing hypoxia, inability to cooperate due to intoxication or impaired mental development, and critical airway compromise in patients who may benefit from tracheostomy (maxillofacial fractures for nasal intubation) [11,12]. Complications from awake fiberoptic intubation have ranged from mucus plugging, discovery of cuff leaks after intubation, inadvertent extubation, and multiple intubation attempts to desaturation (due to bleeding, hypoxia from oversedation, and further trauma and possible edema in the supraglottis which may pose airway obstruction) [12]. Limitations for this technique include experience, training, and skill of the proceduralist performing the intubation.

Given its success rate, awake fiberoptic intubation is the gold standard technique for difficult intubation in patients with massive ameloblastoma with intraoral involvement [2]. Successful awake fiberoptic intubation in cases of massive ameloblastomas has been reported in the literature with the additional use of finger-guided intubations and use of bowed type of Magill forceps to manipulate the ETT [2,15]. Fiberoptic intubation can be done orally or nasally. In our case, nasal intubation was performed given the severe mouth opening limitation, strong gag reflex, and the surgical approach [15]. Nasal intubation risks epistaxis and trauma to nasal turbinates [15].

In a review of the management of 68 cases of ameloblastomas in Nigeria, researchers found that most of the patients had general anesthesia administered using blind nasotracheal intubation (66%), orotracheal intubation, followed by fiberoptic laryngoscopy (11.8%) [16]. Fomete et al. have reported the use of local anesthesia for smaller-sized ameloblastomas ranging under 40mm [16]. Given the size of our patient’s ameloblastoma, a surgical approach to these lesions using local anesthesia was not recommended.

Post-procedure, morbidity and mortality in ameloblastoma resection cases have been mainly associated with asphyxia due to tongue fall and postoperative infections [8]. Anchoring the tongue can allow for better breathing and ease with eating. Jaw thrusts also cannot be applied effectively postoperatively due to a newly reconstructed mandible [14]. Edema of the airway can compound the abovementioned problems and poses a risk to a patent airway. To avoid these problems, it is sometimes preferred to keep patients intubated in the postoperative period. Prophylactic tracheostomy may be safer especially if oral edema is significant and the possibility for airway obstruction persist.

## Conclusions

Large ameloblastomas pose significant difficulty for managing the airways. We present a case of successful awake fiberoptic intubation in a patient with a large ameloblastoma. Awake fiberoptic intubation is a gold standard for large intraoral masses and anesthesiologists should have a low threshold for using this technique in these cases.
